# Antifungal Susceptibility Testing Experience in the Management of Culture-Positive Mucormycosis: Observation from a Large Healthcare System

**DOI:** 10.3390/jof12010034

**Published:** 2026-01-01

**Authors:** Maryam Naveed, Tirdad T. Zangeneh, Nathan P. Wiederhold, William Lainhart, Mohanad M. Al-Obaidi

**Affiliations:** 1 Division of Infectious Diseases, University of Arizona College of Medicine, Tucson, AZ 85724, USAwilliam.lainhart@bannerhealth.com (W.L.); 2 Department of Pathology and Laboratory Medicine, University of Texas Health Science Center at San Antonio, San Antonio, TX 78229, USA; wiederholdn@uthscsa.edu; 3 Department of Pathology and Laboratory Medicine, University of Arizona College of Medicine, Tucson, AZ 85724, USA; 4 Division of Infectious Diseases, Department of Internal Medicine, University of Nebraska Medical Center, Omaha, NE 68198, USA

**Keywords:** antifungal susceptibility testing, mucormycosis, antifungals

## Abstract

**Background:** Mucormycosis, an invasive fungal infection with high morbidity and mortality rates, requires prompt surgical and antifungal therapies; however, the role of antifungal susceptibility testing (AFST) in clinical management of mucormycosis remains underexplored. We aimed to describe the experience of using AFST in the clinical management of mucormycosis. **Methods:** We conducted a retrospective study from 1 October 2017 to 8 February 2023. We included non-pregnant patients aged ≥ 18 years old with a positive culture for Mucorales and with proven or probable mucormycosis. We collected clinical and microbiological data using a chart review. **Results:** Over the study period, a total of 119 patients were included, with 36 (30%) undergoing AFST. Of all patients, the median age was 54 years, with 80 (67%) being White and not Hispanic and 73 (61%) being male. Fifty-three (45%) patients had DM, 27 (23%) had hematological malignancy, 15 (13%) had SOT, and 23 (19%) had COVID-19. Half of the cases met the criteria of proven invasive mucormycosis, with pulmonary involvement being the most common presentation (46, 39%), followed by rhino-cerebral-orbital involvement (35, 29%). The majority of Mucorales isolates were *Rhizopus* species (79, 66%). Among the 36 who underwent AFST, posaconazole minimal inhibitory concentrations (MICs) were lower than isavuconazole (range 0.03 to 2 µg/mL versus 0.1 to 16 µg/mL, respectively). AFST resulted in a change in antifungal therapy from isavuconazole to posaconazole in 3/36 (8%) cases. There was no statistically significant difference in the mortality between the patients whose isolates received AFST versus those who did not have AFST performed. **Conclusions:** AFST led to a change in antifungal therapy in a minority of mucormycosis cases. Further studies to understand the epidemiological range of antifungal MICs and the effect of AFST-informed antifungal therapy are needed.

## 1. Introduction

Mucormycosis, an invasive and often fatal fungal infection, has been a global concern, affecting patients worldwide [1]. In 2022, the World Health Organization (WHO) recognized mucormycosis as one of the high-priority fungal pathogens, as highlighted in their fungal pathogen priority report [2]. Various molds in the order of Mucorales are known to cause mucormycosis, and the most common pathogens are *Rhizopus* spp., *Mucor* spp., *Lichtheimia* spp., and *Rhizomucor* spp. [3,4]. Mortality can be >50% in severe cases and in patients with an underlying risk of severe immunosuppressive conditions [1]. Several risk factors contribute to the development of mucormycosis, which can influence clinical outcomes such as uncontrolled diabetes mellitus, neutropenia due to hematological malignancy (HM) or after allogeneic hematopoietic stem cell transplant (HSCT), and solid organ transplant (SOT) [1].

The management of mucormycosis necessitates a comprehensive, multidisciplinary strategy and the administration of antifungals [5]. The antifungal arsenal for mucormycosis treatment is limited, with liposomal amphotericin B (L-AMB) the most efficacious agent, which is recommended by current guidelines, albeit with a notable risk of nephrotoxicity [5]. Current triazole antifungals, including posaconazole and isavuconazole, exhibit in vitro and in vivo activity against Mucorales [4,6,7]. They both act on the inhibition of ergosterol biosynthesis, which results in toxic sterol accumulation and cell death [5,8]. However, their effectiveness can be species-specific, as posaconazole has demonstrated reduced efficacy in murine models infected with *Mucor circinelloides* [9]. Isavuconazole was approved by the United States Food and Drug Administration (USFDA) for mucormycosis treatment in 2015 [8]. Posaconazole, despite lacking USFDA approval for mucormycosis, is frequently utilized as a step-down therapy [5]. Given the lack of adopted interpretable breakpoints by the European Committee on Antimicrobial Susceptibility Testing (EUCAST) and Clinical and Laboratory Standards Institute (CLSI) [10,11], the role of antifungal susceptibility testing (AFST) in the management of mucormycosis remains unclear, and current expert opinion does not recommend routine testing [5]. In our practice, we observed that ordering of AFST against Mucorales isolates in cases of mucormycosis is not infrequent; therefore, we aimed to describe AFST usage patterns and to assess the impact of AFST on the clinical decision-making process for mucormycosis management.

## 2. Methods

This is a retrospective cohort study including patients from a large hospital network (20 hospitals) in the state of Arizona, in the United States.

**Figure 1:**
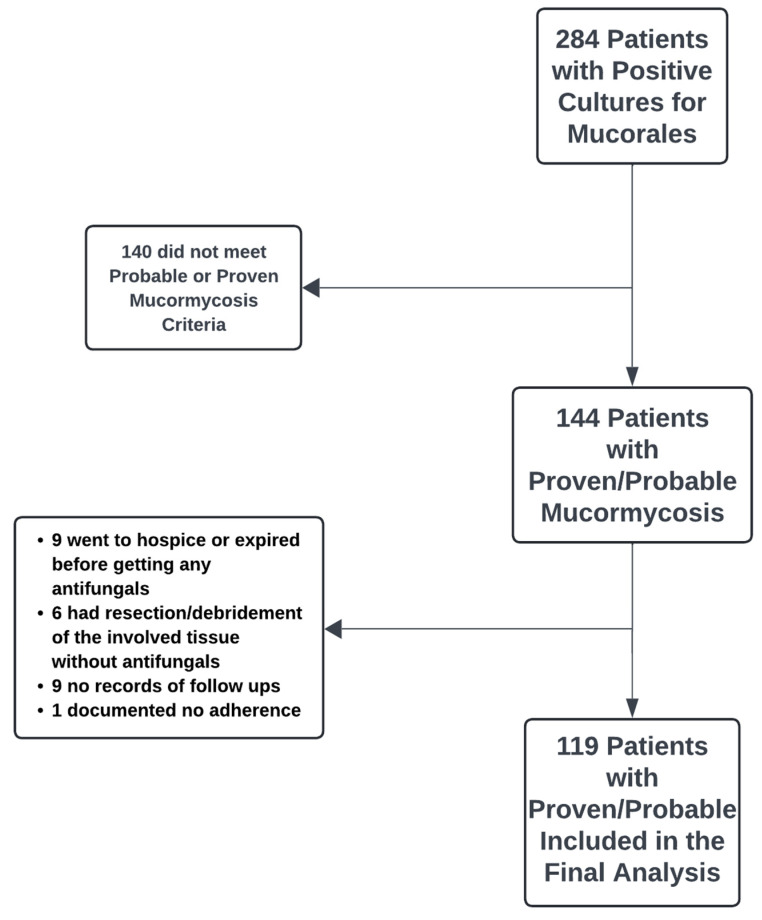
Patients included in the study’s final analysis. Criteria for proven mucormycosis require positive clinical and radiological findings and positive histopathology, and for probable cases, they are required to have positive clinical and radiological findings in the presence of positive culture with Mucorales.

Patients with culture-positive Mucorales species from 1 October 2017 to 8 February 2023 were evaluated, and non-pregnant patients ≥ 18 years old were included. Cases of mucormycosis meeting the European Organization for Research and Treatment of Cancer and the Mycoses Study Group Education and Research Consortium (EORTC/MSGERC) definition for probable or proven mucormycosis [12] were included. The study followed Strengthening the Reporting of Observational Studies in Epidemiology (STROBE) guidelines (supplements). For COVID-19-associated mucormycosis, cases meeting the criteria of proven disease are the same as the EORTC/MSG criteria [12]. Since EORTC/MSG criteria did not have COVID-19 as an underlying risk factor, we borrowed the definition of probable mucormycosis in patients who received treatment with steroids for COVID-19, had a positive culture for Mucorales, and had clinical and radiological findings consistent with mucormycosis [13,14]. Patients were excluded if they were discharged to hospice or died and had received ≤2 days of antifungal therapy. Details of patient selection can be seen in Figure 1. Each included patient’s chart was reviewed, and demographics, clinical data, including risk factors (diabetes mellitus [DM] and immunosuppressive condition), mucormycosis site, antifungal therapy, microbiological data, and clinical outcomes with a follow-up period of 24 weeks, and data were entered into Research Electronic Data Capture (REDCap).

Fungi were grown using standard mycological media, and morphological identification was performed to identify different genera. Fungal cultures in our laboratories use a combination of Sabouraud Dextrose Agar, Inhibitory Mold Agar, Brain Heart Infusion Agar (with or without Gentamicin and Chloramphenicol), Mycobiotic Agar, and Sabouraud Dextrose Broth, depending on the specimen source. All fungal culture plates are incubated in ambient air at 25–30 °C. AFST was performed at the Fungus Testing Laboratory at the University of Texas Health Science Center at San Antonio upon the physician’s request. AFST was performed using broth microdilution according to the Clinical and Laboratory Standards Institute (CLSI) M38 standard (CLSI M38Ed3, 2017) [15]. The final concentrations for amphotericin B, posaconazole, and isavuconazole ranged from 0.03 to 16 µg/mL. MICs, indicating 100% inhibition of growth, were visually determined after 24 h of incubation at 35 °C [4], as it was previously described by Badali et al. and the CLSI M38 broth microdilution method [4,15].

The primary objective of this study was to describe the patients’ population characteristics that had AFST ordered vs. those that did not have AFST ordered and assess the effect of AFST on the clinical decision-making of mucormycosis antifungal choice. The secondary objective was to compare the antifungal MICs of different Mucorales species.

### Statistical Analysis

We utilized descriptive statistics with Pearson’s χ^2^ or Fisher’s exact tests to assess statistical differences between categorical variables and with Student’s *t*-test, Wilcoxon’s rank, ANOVA, and Kruskal–Wallis tests to assess statistical differences between continuous variables, as indicated. Demographic, clinical, and microbiological characteristics were compared between the AFST and non-AFST groups. The AFST presented as MICs in µg/mL were presented with median (range), mode, and geometric mean (GM). The study is meant to describe the clinical experience of AFST; therefore, we did not calculate the sample size.

Two-tailed tests with *p* < 0.05 were considered statistically significant. Statistical analysis was performed using R version 4.4.2 (31 October 2024).

## 3. Results

During the study period, 119 patients with positive cultures for Mucorales met the criteria of invasive mucormycosis, with 36 (30%) undergoing AFST. Of all the patients, the median age was 54 (interquartile range [IQR] 42–64), the majority (80 [67%]) were White Not Hispanic, and 73 (61%) were male. Most cases had pulmonary disease (46, 39%), rhino-cerebral-orbital (ROC) 35 (29%), and the remainder were disseminated (12, 10%) or had soft tissue involvement (26, 22%). Half of the cases, 59 (50%), met criteria for proven invasive mucormycosis, with most cases of proven mucormycosis involving sinus disease. In most cases, 53 (45%) had DM, 27 (23%) had hematological malignancy, 15 (13%) had SOT, and 23 (19%) had COVID-19. Twenty-nine patients with HM or SOT were receiving antifungal prophylaxis with fluconazole (12, 41%), posaconazole (11, 38%), voriconazole (4, 14%), and isavuconazole (2, 7%). There were no statistically significant differences between AFST and non-AFST groups in regard to antifungal prophylaxis. For the antifungal treatment of mucormycosis, most patients were started on liposomal amphotericin B 85 (71%), with an initial triazole, including isavuconazole (45, 38%) or posaconazole (36, 30%). The crude mortality at 24-week follow-up was 59/119 (50%), with no statistically significant difference between AFST and non-AFST groups, as seen in Table 1.

### Microbiology

Thirty-six (30%) patients with mucormycosis had AFST performed. Patients in the AFST group were more commonly initiated on L-AMB (31, 86%) versus 54 (65%) patients in the non-AFST group (*p*-value = 0.02), and more frequently in the SOT group. Besides those mentioned above, the two groups had no other statistically significant differences (Table 1).

Among all the isolates in this study, *Rhizopus* spp. (79, 66%, 3 identified by PCR as *R. arrhizus*), *Mucor* spp. (31, 26%, one identified by PCR as *M. circinelloides* and one as *Actinomucor elegans*), *Lichtheimia* spp. (6, 5%), and one of each of the following species: *Syncephalastrum* sp., *Cunninghamella* sp., and *Apophysomyces* sp. The AFST group included *Rhizopus* spp. (26, 72%), and *Mucor* spp. (4, 11%), whereas the non-AFST group included 53 *Rhizopus* spp. (64%) and 27 *Mucor* spp. (33%, *p*-value = 0.008), as seen in Table 1.

Of the 36 clinical isolates with AFST, 36 (100%) had posaconazole MICs, 32 had isavuconazole MICs (89%), and 29 had amphotericin B MICs (81%). The posaconazole MIC across all Mucorales in this study ranged from 0.03 to 2 µg/mL with a GM of 0.25 µg/mL and modes of 0.125 µg/mL and 0.25 µg/mL (bimodal); the isavuconazole MIC ranged from 0.1 to 16 µg/mL with a GM of 1.5 µg/mL and mode of 2 µg/mL; and the amphotericin B MIC ranged from 0.03 to 4 µg/mL, with a GM of 0.16 µg/mL and mode of 0.125 µg/mL, as seen in Table 2.

In three cases, AFST resulted in a change in the antifungal therapy from isavuconazole to posaconazole because of higher isavuconazole MICs versus posaconazole MICs. There was a statistically significant difference in the posaconazole GM MIC between the group that had an antifungal therapy change versus the group that did not have therapy changed, 0.794 µg/mL vs. 0.214 µg/mL, *p*-value = 0.025, respectively, as seen in Table S1. The three cases had the following Mucorales isolates with the respective posaconazole and isavuconazole MICs: *Apophysomyces* sp., 1 µg/mL, 4 µg/mL; *Mucor* sp., 1 µg/mL, 4 µg/mL; and *Rhizopus* sp., 0.5 µg/mL, 2 µg/mL. Detailed AFST is included in Table S2.

## 4. Discussion

In this study, we present real-world clinical experience in managing culture-proven mucormycosis with a subset of cases that had AFST. While antifungal MICs varied, the results did not lead to a change in antifungal therapy in the majority of the patients.

A comparison of clinical characteristics between the AFST and non-AFST groups shows that SOT recipients underwent AFST more frequently than the non-AFST cohort, likely due to the availability of resources and specialized care at some specific centers. This trend can also be seen in the higher utilization of L-AMB in the AFST group. While our study observed a high crude mortality rate of 50% at the 24-week follow-up, there was no statistically significant difference between the AFST and non-AFST groups, suggesting little effect of AFST on patient outcomes. However, evaluating the impact of AFST on mortality in our cohort is limited by the heterogeneity of different risk groups and unaccounted confounders.

Unlike invasive yeast infections, such as those caused by *Candida* spp., the experience of utilizing Mucorales AFST in clinical management is limited. The European Committee on Antimicrobial Susceptibility Testing (EUCAST) provides guidance with antifungal breakpoints for *Aspergillus* spp., and CLSI provides guidance for voriconazole and isavuconazole against *Aspergillus fumigatus*; however, there is no breakpoint for any antifungal against members of the order of Mucorales by either EUCAST or CLSI [10,11]. Therefore, this renders any interpretation of Mucorales MICs in the clinical settings uncertain. Most in vitro studies of available antifungals for the use of human fungal infections have reported on the activity of amphotericin B, posaconazole, and isavuconazole against mucoralean fungi [4,16]. However, the in vitro data from different Mucorales have resulted in a range of MICs that may be species- or genus-specific. In most cases, the isavuconazole MICs were shown to be 2–4-fold higher compared to posaconazole. Isavuconazole MICs tend to be very high (MICs of ≥16 µg/mL) for *Mucor circinelloides* and *Syncephalastrum* spp. [4,17,18], which raises concerns for clinical failure against pathogen-specific mucormycosis. In contrast, posaconazole appears to have good in vitro activity against most Mucorales with a GM MIC of ≤2 µg/mL, except for *Cunninghamella bertholletiae* with a higher GM MIC of 7 µg/mL [16]. In our study, most of the isolates in the AFST group were of *Rhizopus* spp., and similar to the previous in vitro observations, the posaconazole MICs were lower than isavuconazole by more than 4-fold. The higher isavuconazole MICs resulted in antifungal therapy change among the three cases in the AFST group in our study. However, early and effective antifungal therapy is crucial early in the course of mucormycosis. Understanding different Mucorales species corresponding to antifungal susceptibility patterns can help guide better treatment choices early in the disease course. Therefore, species identification using molecular methods and understanding the molecular mechanisms of various antifungal resistances can be instrumental in selecting appropriate antifungal therapy [19].

The strengths of our study include a relatively large sample size of proven and probable mucormycosis cases with positive cultures, with a moderate-sized AFST cohort, helping provide real-world insight into the utility of Mucorales AFST in clinical settings. The study implemented a detailed chart review to evaluate the clinician’s decision to change antifungals based on AFST results.

The limitations of the study include the retrospective design with selection bias of patients and the lack of AFST for all azoles. Although the study’s design and methodology largely adhered to STROBE guidelines, no sample size calculation was performed due to its retrospective and descriptive nature. Therefore, the evaluation of the AFST effect on overall survival was underpowered, and this conclusion is limited. Also, AFST might not have affected the clinical decision early in the course of the disease management due to delays in obtaining samples and AFST results. Moreover, we lack species-level identification, which restricts our ability to draw conclusions about the differences in AFST among different species. Additionally, since these data include patients from many medical centers within our healthcare system. Thus, an additional possible confounder is the effect of AFST use in centers supported by a higher level of care. Moreover, antifungal efficacy analysis on the survival is not within the scope of this study, as we have not included the measurement of antifungal starting time, dose of the antifungals, and timing of surgical intervention.

In conclusion, our study demonstrates that although AFST was utilized to guide clinical decisions, it resulted in changes to antifungal therapy in a minority of cases. Early knowledge of species-specific MICs, especially for certain Mucorales species, can help inform antifungal choices when paired with rapid identification methods. Future prospective studies are warranted to further elucidate the potential benefits of AFST in the management of mucormycosis.

## Figures and Tables

**Table 1:** Clinical characteristics of cases that underwent AFST versus non-AFST.

Characteristic	Non-AFST *n* = 83 ^1^	AFST *n* = 36 ^1^	*p*-Value ^2^
Age	55 (44, 66)	53 (41, 63)	0.3
Race/Ethnicity			0.6
Black	3 (3.6%)	1 (2.8%)	
Hispanic	21 (25%)	13 (36%)	
Other	1 (1.2%)	0 (0%)	
White Not Hispanic	58 (70%)	22 (61%)	
Disease Type			0.6
Probable	43 (52%)	17 (47%)	
Proven	40 (48%)	19 (53%)	
Surgery	44 (53%)	25 (69%)	0.10
ROC	22 (27%)	13 (36%)	0.3
Pulmonary	34 (41%)	12 (33%)	0.4
Disseminated	8 (9.6%)	4 (11%)	0.8
Skin and Soft Tissue	19 (23%)	7 (19%)	0.7
COVID-19	18 (22%)	5 (14%)	0.3
DM	38 (46%)	15 (42%)	0.7
DKA	15 (18%)	3 (8.3%)	0.2
CKD	10 (12%)	6 (17%)	0.6
Cirrhosis	4 (4.8%)	2 (5.6%)	>0.9
Hematological Malignancy	21 (25%)	6 (17%)	0.3
SOT	7 (8.4%)	9 (25%)	0.021
Other Immunosuppression *	4 (4.8%)	3 (8.3%)	0.4
Initial Posaconazole	24 (29%)	12 (33%)	0.6
Initial Isavuconazole	27 (33%)	18 (50%)	0.071
Initial L-AMB	54 (65%)	31 (86%)	0.020
Fungus Genus			0.008
*Apophysomyces* sp.	0 (0%)	1 (2.8%)	
*Cunninghamella* sp.	0 (0%)	1 (2.8%)	
*Lichtheimia* spp.	3 (3.6%)	3 (8.3%)	
*Mucor* spp.	27 (33%)	4 (11%)	
*Rhizopus* spp.	53 (64%)	26 (72%)	
*Syncephalastrum* sp.	0 (0%)	1 (2.8%)	
Antifungal Prophylaxis			0.2
Fluconazole	6 (7.2%)	6 (17%)	
Isavuconazole	1 (1.2%)	1 (2.8%)	
Posaconazole	10 (12%)	1 (2.8%)	
Voriconazole	2 (2.4%)	2 (5.6%)	
None	64 (77%)	26 (72%)	
24-Week Mortality	44 (53%)	15 (42%)	0.3

^1^ Median (Q1, Q3); *n* (%); ^2^ Wilcoxon rank sum test; Fisher’s exact test; Pearson’s chi-squared test, where appropriate; AFST, antifungal susceptibility test; ROC, rhino-cerebral-orbital; COVID-19, coronavirus disease 2019; DM, diabetes mellitus; DKA, diabetic ketoacidosis; CKD, chronic kidney disease; SOT, solid organ transplantation; L-AMB, liposomal Amphotericin B. * Other immunosuppression (4 received autoimmune disease, one received high-dose steroid use in renal cell cancer with metastasis to the brain, and one received high-dose steroids for influenza).

**Table 2:** Minimal inhibitory concentrations in µg/mL for amphotericin B and triazoles among clinical Mucorales isolates.

Antifungals (*n*)	MIC 50	MIC 90	Modal	GM
Posaconazole (36)	0.25	1.00	0.125 and 0.250 (bimodal)	0.249
Isavuconazole (32)	2.0	8	2	1.507
Amphotericin B (29)	0.13	1	0.125	0.165

*n*, number of isolates tested; MIC, minimal inhibitory concentration; GM, geometric mean.

## Data Availability

The data presented in this study are available on request from the corresponding author due to participating healthcare institution privacy policies which prohibit public disclosure of patients related information to maintain patients’ confidentiality.

## Supplementary Materials

The following supporting information can be downloaded at: https://www.mdpi.com/article/10.3390/jof12010034/s1, Table S1: Antifungal MICs geometric means for patients who had antifungals changed based on AFST result versus the ones who did not have the antifungals change; Table S2: MICs for amphotericin B and triazoles among clinical Mucorales isolates.
