# Supplementary material for: Antifungal Susceptibility Testing Experience in the Management of Culture-Positive Mucormycosis: Observation from a Large Healthcare System
Source: J Fungi (Basel). 2026 Jan 1;12(1):34. doi: 10.3390/jof12010034 (PMC12842725; doi:10.3390/jof12010034)
Supplement: Supplementary file 1 [file jof-12-00034-s001.zip › jof-4071365-supplementary.pdf]

Table S1. Antifungal MICs geometric means for patients who had antifungals changed based on AFST result versus the ones who did not have the antifungals change.

	Antifungal Change		
**Antifungals**	**No** N = 32[1]	**Yes** N = 3[1]	**p-value**[2]
Posaconazole GM	0.214	0.794	0.025
Isavuconazole GM	1.31	3.170	0.12
Amphotericin B GM	0.180	0.766	0.11

[1] Geometric Mean

[2] Wilcoxon rank sum test

AFST, antifungal susceptibility test; MICs, minimal inhibitory concentrations.

Table S2. MICs for amphotericin B and triazoles among clinical Mucorales isolates.

Fungus	Amphotericin B MICs			Posaconazole MICs			Isavuconazole MICs		
	Median (range)	Mode	GM	Median (range)	Mode	GM	Median (range)	Mode	GM
Rhizopus spp. N = 26	0.13 (0.03, 4)	0.125	0.195	0.25 (0.03, 1)	0.125	0.212	1.0 (0.1, 16)	1	1.233
Mucor spp. N = 4	0.09 (0.03, 0.13)	0.125	0.728	0.31 (0.03, 1)	0.125	0.208	2.5 (0.5, 4)	4	1.682
Lichtheimia spp. N = 3	0.13 (0.06, 0.25)		0.123	0.13 (0.13, 2)	1	0.315	2 (2, 2)	2	2
Apophysomyces sp. N = 1	0.06		0.06	1		1	4		4
Syncephalastrum sp. N = 1				2		2	16		16
Cunninghamella sp. N = 1	1		1	0.5		0.5			

MIC, minimal inhibitory concentration; GM, geometric mean.
